# Engineering of TIMP‐3 as a LAP‐fusion protein for targeting to sites of inflammation

**DOI:** 10.1111/jcmm.14019

**Published:** 2018-11-18

**Authors:** Ben M. Alberts, Sandra M. Sacre, Peter G. Bush, Lisa M. Mullen

**Affiliations:** ^1^ Brighton and Sussex Medical School University of Sussex Brighton UK; ^2^ Pharmacy and Biomolecular Sciences University of Brighton Brighton UK

**Keywords:** latency‐associated peptide, matrix metalloproteinase, osteoarthritis, recombinant TIMP‐3

## Abstract

Tissue inhibitor of metalloproteinase (TIMP)‐3 is a natural inhibitor of a range of enzymes that degrade connective tissue and are involved in the pathogenesis of conditions such as arthritis and cancer. We describe here the engineering of TIMP‐3 using a novel drug‐delivery system known as the ‘LAP technology’. This involves creating therapeutic proteins in fusion with the latency‐associated peptide (LAP) from the cytokine TGF‐? to generate proteins that are biologically inactive until cleavage of the LAP to release the therapy. LAP‐TIMP‐3 was successfully expressed in mammalian cells and the presence of the LAP resulted in a 14‐fold increase in the quantity of recombinant TIMP‐3 produced. LAP‐TIMP‐3 was latent until release from the LAP by treatment with matrix metalloproteinase when it could inhibit proteases of the adamalysins and adamalysins with thrombospondin motifs families, but not matrix metalloproteinases, indicating that this version of TIMP‐3 is a more specific inhibitor than the native protein. There was sufficient protease activity in synovial fluid from human joints with osteoarthritis to release TIMP‐3 from the LAP fusion. These results demonstrate the potential for development of TIMP‐3 as a novel therapy for conditions where upregulation of catabolic enzymes are part of the pathology.

## 1 INTRODUCTION

Two families of endopeptidases, matrix metalloproteinases (MMPs) and aggrecanases are essential for the turnover of extracellular matrix molecules during tissue remodelling and repair. Their activity is tightly regulated under physiological conditions, but is up‐regulated in inflammatory conditions. Some of the most important endogenous inhibitors of MMPs are the tissue inhibitor of metalloproteinases (TIMPs) which are a family of four inhibitors (TIMP‐1‐4).^1^ TIMP‐3 has the widest inhibitory activity amongst the TIMPs, inhibiting not just the MMPs but many members of the related adamalysins (ADAM)^2^ and adamalysins with thrombospondin motifs (ADAMTSs).^3^ Unfortunately, development of synthetic inhibitors of MMPs has not performed well in clinical trials causing severe toxicity and a musculoskeletal syndrome involving joint pain, oedema, and reduced mobility.^4^ The broad nature of these inhibitors is deemed responsible for these side effects, so any future development of this approach will require more selective inhibitors.^5^, ^6^

**Figure 1:**
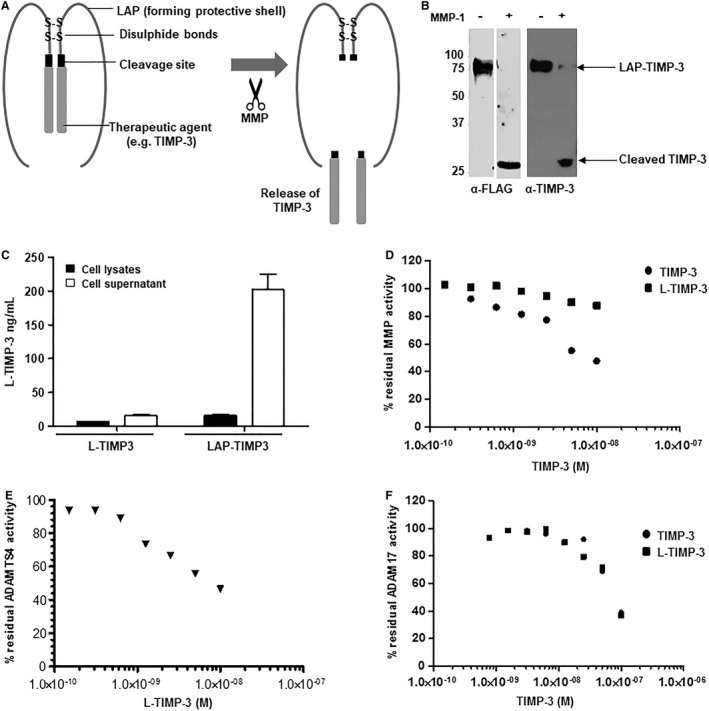
Engineering of LAP‐TIMP‐3. A, The latency‐associated peptide (LAP) from TGF‐β forms a shell around the therapeutic agent, rendering it biologically inactive. A cleavage site specific for matrix metalloproteinase (MMP) is engineered in between the LAP and the therapeutic agent, enabling its release at sites of disease B. Western blot analysis of purified LAP‐TIMP‐3 ‐/+ recombinant MMP‐1. C, Quantification of TIMP‐3 fusion proteins accumulating in the medium of transiently transfected HEK293T cells or in cell lysates measured by TIMP‐3 ELISA. Bars represent the mean ± SE from quadruplicate cultures from three separate experiments D. MMP inhibitory activity of TIMP‐3 or L‐TIMP‐3 assessed by MMP fluorometric assay. E, Inhibition of aggrecanase (ADAMTS‐4) activity by L‐TIMP‐3. Purified L‐TIMP‐3 was incubated with ADAMTS‐4 for 1 h prior to measuring aggrecanase activity using a fluorescent peptide substrate. F, Inhibition of ADAM 17 activity by purified L‐TIMP‐3 measured by ADAM17 fluorometric assay. Data are representative of two (ADAMTS‐4) or three (MMP‐1 and ADAM17) separate experiments performed in duplicate

We have been working for a number of years on a novel drug delivery system, known as the latency‐associated peptide (‘LAP’) fusion protein technology. This strategy is based on creating recombinant therapeutic proteins, such as anti‐inflammatory cytokines, in an inactive (latent) form, so that they can be administered systemically without causing side effects.^7^ This system is based on the naturally occurring LAP from the cytokine transforming growth factor (TGF)‐β. Therapeutic molecules can be engineered with the LAP at the N terminus (the same arrangement as that found in native TGF‐β), so that the expressed protein consists of the recombinant therapeutic agent surrounded by the LAP which prevents interaction with ligands/substrates rendering it biologically inactive (see Figure 1A). These molecules are also engineered with cleavage sites specific for proteases found at sites of inflammation. The LAP technology has a number of advantages: the therapy is given systemically and delivery is targeted to the site of disease; there are no effects on normal immune responses; and much greater doses of the therapeutic agent can be administered to achieve high local concentrations as there will be no systemic effects.^8^ Using this system, we developed a latent (biologically inactive) form of the immunomodulatory cytokine, interferon‐beta (IFN‐β), which has a 40‐fold longer half‐life than native IFN‐β. Latency, therapeutic efficacy and targeting potential of LAP‐IFN‐β fusion proteins have been previously demonstrated in vivo^9^ and a number of cytokines, growth factors, and peptides have successfully been made latent using this approach.^10^, ^11^

The aim of this study is to express TIMP‐3 as a LAP‐fusion protein with an MMP cleavage site and assess the latency of the expressed protein and subsequent biological activity upon MMP cleavage.

## 2 METHODS

Detailed materials and methods are provided in Data S1. LAP‐TIMP‐3 fusion proteins were cloned in pcDNA6 and expressed in HEK 293T cells. Enzyme activity assays for ADAMTS‐4, ADAM17 and MMP as well as a bovine cartilage explant assay were used to assess the biological activity of LAP‐TIMP‐3 before and after incubation with MMP. Synovial fluids and sera from patients undergoing joint replacement surgery for OA were collected after obtaining informed written consent and tested for their ability to cleave LAP‐TIMP‐3.

## 3 RESULTS

### 3.1 Expression of LAP‐TIMP‐3 and cleavage with MMP‐1

Mature human TIMP‐3 was expressed as a LAP‐fusion protein with a truncated cleavage site that was efficiently cleaved by recombinant MMP‐1 (Figure 1B). Cleavage resulted in a residual leucine at the N terminus of the TIMP‐3 released from the LAP. As previous studies had indicated that any additional amino acids substantially altered the biological activity of TIMP‐3,^12^ we also expressed L‐TIMP to determine the effect of this extra N‐terminal amino acid on biological activity. This version of TIMP‐3 was produced in much lower quantities in HEK 293T cells than LAP‐TIMP‐3 (Figure 1C), but when purified, retained inhibitory activity for ADAMTS‐4 (Figure 1D) and ADAM17 (Figure 1E), but not for MMP (Figure 1F).

### 3.2 Biological activity of LAP‐TIMP‐3 after cleavage with MMP‐1

**Figure 2:**
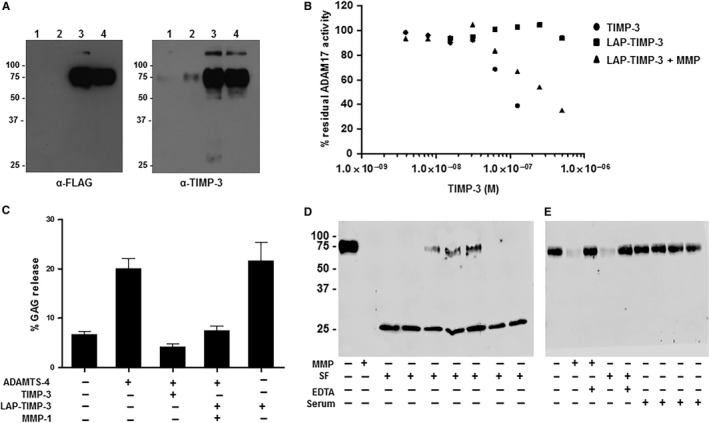
Cleavage of LAP‐TIMP‐3 releases biologically active TIMP‐3. A, Western blot of various fractions from affinity purification LAP‐TIMP‐3 via the N‐terminal His‐tag using immobilized metal affinity purification. Lane 1 – Unbound fraction. Lane 2 ‐ 1M NaCl wash. Lane 3– 50 mmol/L imidazole. Lane 4 – 300 mmol/L imidazole. B, Effect of MMP cleavage of inhibitory activity of LAP‐TIMP‐3 for ADAM17 measured by ADAM17 inhibition assay. Data are representative of two separate experiments performed in duplicate. C, LAP‐TIMP‐3 ‐/+ MMP‐1 was incubated with ADAMTS‐4 prior to addition to bovine cartilage explants. The percentage GAG release was measured by DMMB assay 3 days later. Bars represent the mean ± SE of three independent experiments (*n*=6). D, Cleavage of LAP‐TIMP‐3 by synovial fluids from patients with OA analysed by Western blotting using anti‐FLAG antibody. E, Western blot analyses using anti‐FLAG antibody of LAP‐TIMP‐3 incubated with SF in the presence of EDTA or with sera from patients with OA. D and E, MMP ‐ recombinant MMP used as a positive control

LAP‐TIMP‐3 was purified from HEK 293T cells via the N‐terminal His‐tag by immobilized metal affinity chromatography (Figure 2A) with a final yield of about 150 μg from 1 L of HEK conditioned media. Upon cleavage with MMP‐1, the TIMP‐3 released from the molecule retained its ability to inhibit ADAM17 (Figure 2B) and ADAMTS‐4 as assessed by cartilage explant assay (Figure 2C). An important consideration was whether there is sufficient MMP activity in the osteoarthritic joint to efficiently cleave the LAP‐TIMP‐3 molecule. To test this, we collected synovial fluid samples from patients with osteoarthritis and incubated the purified LAP‐TIMP‐3 with these fluids. LAP‐TIMP‐3 was completely cleaved by ten of the thirteen synovial fluids tested, with the remaining three synovial fluids cleaving about 85% of the LAP‐TIMP‐3 (Figure 2D, Figure S1). Sera from OA patients did not cleave the LAP‐TIMP‐3 and cleavage was also inhibited by addition of EDTA to synovial fluids or to MMP (Figure 2E).

## 4 DISCUSSION

We report here a novel method of producing recombinant TIMP‐3 in a mammalian cell culture system that can also be used to deliver the protein to sites of disease. The presence of the LAP drastically increased the quantity of TIMP‐3 that could be purified. This increased yield of LAP‐TIMP‐3 compared with that reported by other groups may also be a consequence of using the His‐tag on the N terminus of the fusion protein for purification. Previous studies relied on the C‐terminal FLAG tag on TIMP‐3 which is subject to substantial processing and decreased overall yields of purified material.^13^ However, there was also much greater quantities of TIMP‐3 accumulating in the medium when expressed as a LAP‐fusion protein and we hypothesize that this could be a consequence of LAP preventing interactions with cellular receptors and subsequent endocytosis; indeed it is this function that makes it possible to produce latent molecules.^8^ Consistent with this hypothesis is our observation that LAP‐fusions of molecules whose ligands are soluble are not latent (Figure S2).

As expected, TIMP‐3 was a more specific inhibitor when produced as a LAP‐fusion protein because of the residual leucine residue on the N terminus after cleavage with MMP. This is consistent with previous work on N‐terminal mutants of TIMP‐3 where the addition of an N‐terminal alanine residue was sufficient to completely abrogate the inhibitory activity for MMPs, but retained the ability to inhibit aggrecanases.^12^ This actually makes the prospect of LAP‐TIMP‐3 as a therapeutic more appealing. Clinical trials with inhibitors of MMPs have generally been disappointing because of adverse side effects which are attributed to the broad spectrum of the inhibitors tested. Greater specific activity could circumvent these problems previously encountered with the broad‐spectrum MMP inhibitors^6^ and could be used alongside existing therapies to improve clinical outcome.

An important finding of this study is that MMP cleavage of LAP‐TIMP‐3 was essential for biological activity of the molecule as shown in both the in vitro enzyme assays and in the cartilage degradation assay. The LAP technology is designed to allow systemic administration of the latent protein into the circulation and subsequent cleavage and accumulation at sites of inflammation via cleavage by MMPs. Therefore, our finding that there is sufficient MMP activity in OA synovial fluid to cleave LAP‐TIMP‐3, but not in OA patient serum, is important. These data widen the therapeutic possibilities of LAP‐TIMP‐3, particularly as ADAM17 which contributes to pro‐inflammatory TNF activity^14^ was also inhibited by TIMP‐3 when released from the LAP.

## CONFLICT OF INTEREST

The authors confirm that there are no conflicts of interest.
