# Supplementary figures and images for: Engineering of TIMP‐3 as a LAP‐fusion protein for targeting to sites of inflammation
Source: J Cell Mol Med. 2018 Nov 18;23(2):1617–21. doi: 10.1111/jcmm.14019 (PMC6349231; doi:10.1111/jcmm.14019)

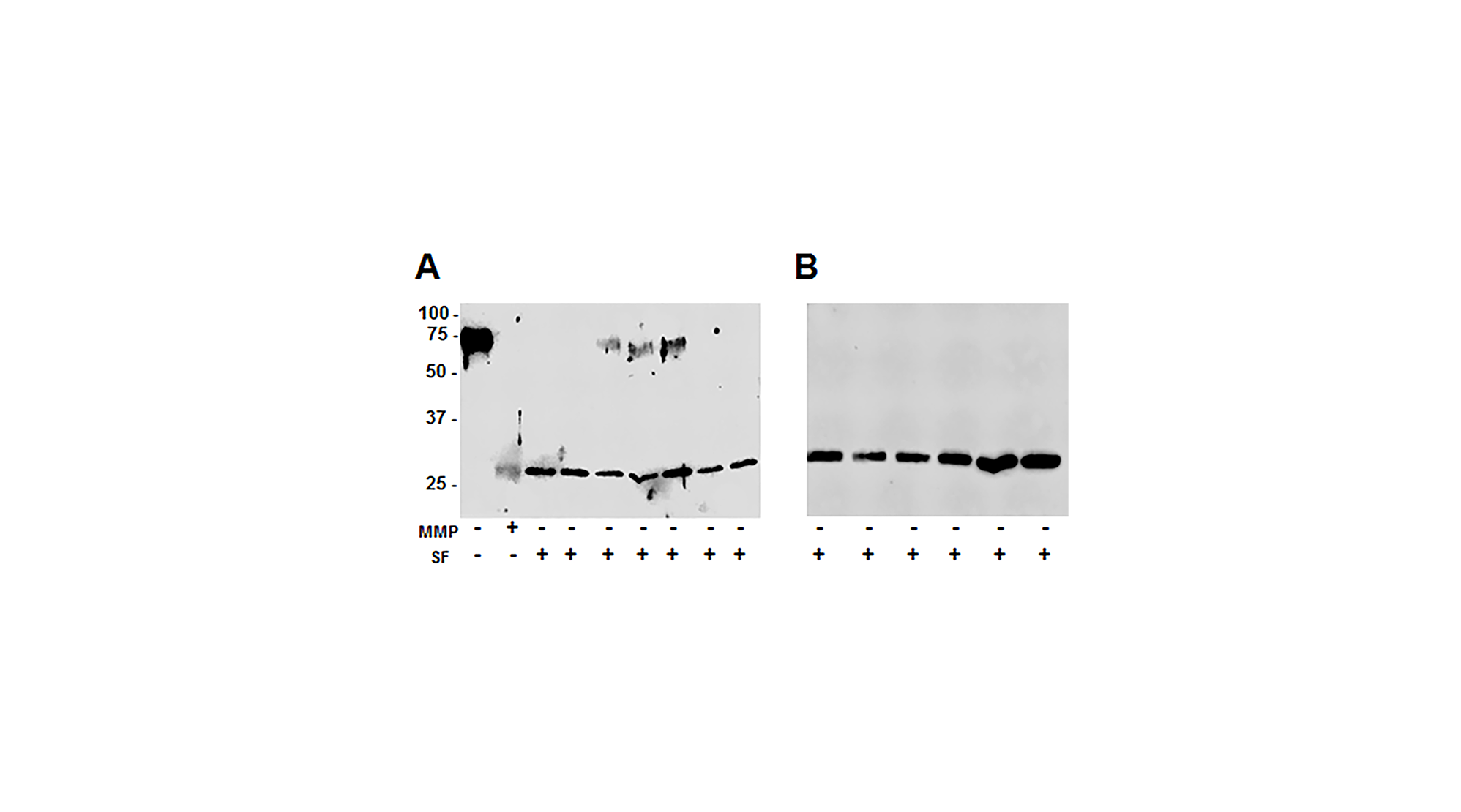

Supplement: Supplementary file 1 [file JCMM-23-1617-s001.tif]

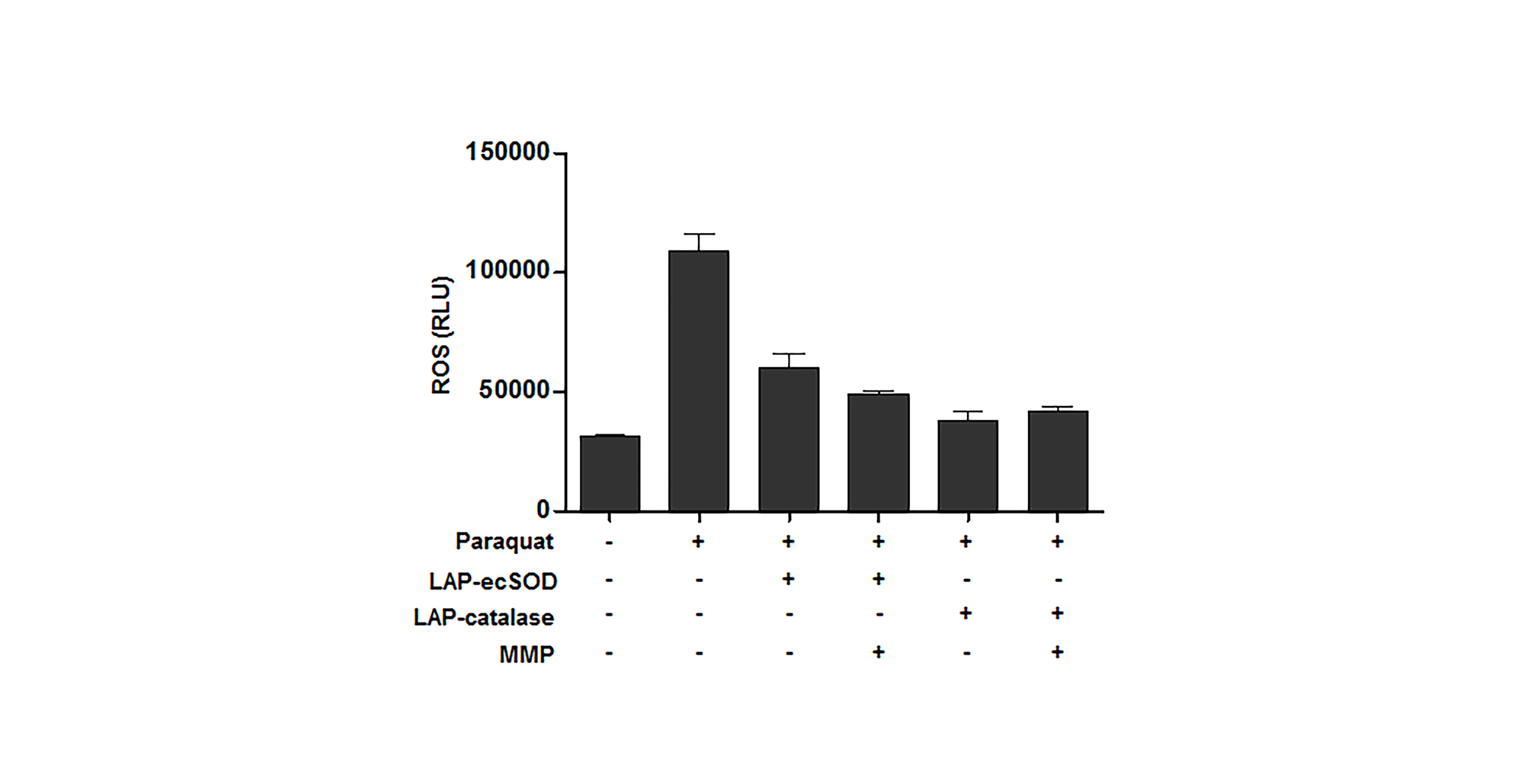

Supplement: Supplementary file 2 [file JCMM-23-1617-s002.tif]
