# Supplementary material for: Engineering of TIMP‐3 as a LAP‐fusion protein for targeting to sites of inflammation
Source: J Cell Mol Med. 2018 Nov 18;23(2):1617–21. doi: 10.1111/jcmm.14019 (PMC6349231; doi:10.1111/jcmm.14019)
Supplement: Supplementary file 3 [file JCMM-23-1617-s003.docx]

**Supplementary Material 1**

**Materials and Methods**

**Reagents**

Cell culture reagents used were penicillin-streptomycin, DMEM and fetal calf serum (FCS) from Sigma (Dorset, UK) and serum-free OPTI-MEM from Fisher Scientific (Loughborough, UK). Recombinant human TIMP-3 and human ADAMTS-4 were purchased from R&D systems (Abingdon, Oxon, UK). Dimethylmethylene blue (DMMB) reagent and shark chondroitin sulphate were purchased from Sigma.

**Synovial fluid**

Synovial fluid was collected from patients (age range: 48-86 years) undergoing either hip (*n*=5) or knee (*n*=8) joint replacement surgery for osteoarthritis. All patients gave written informed consent and the study was approved by the Wales REC7 Research Ethics Committee, REC number: 14/WA/1016. Synovial fluid samples were centrifuged to remove cells and debris and stored at -20°C until further use.

**Cloning of human TIMP-3 in pcDNA6**

LAP-TIMP-3 was cloned in pcDNA6 (Invitrogen, Life Technologies, Paisley, UK) using the LAP-MMP-IFN-β construct previously described^[^[^1^](#_ENREF_1)^]^ and to which we subsequently added a HIS-tag at the N-terminus of the LAP by PCR cloning. In this construct, LAP-MMP-IFN-β is cloned downstream of the IL-2 signal sequence to ensure efficient secretion of the recombinant protein. The sequence coding for mature mouse LAP plus the MMP cleavage sequence was amplified from LAP-MMP-IFN-β using a forward primer complementary to HIS-tagged mouse LAP (Table S1, primer 1) and a reverse primer (Table S1, primer 2) to generate a PCR product coding for LAP-MMP with the truncated MMP cleavage sequence PLGL. Mature human TIMP-3 with a C-terminal FLAG-tag was amplified by PCR from the expression vector TIMP-3-FLAG/pCEP4^[^[^2^](#_ENREF_2)^]^ (a generous gift from Dr. Linda Troeberg, University of Oxford) using specific primers designed to add overlapping sequences from the 3‘ end of the LAP-MMP construct described above (Table S1, primers 3 and 4). Stitching PCR was then used to generate LAP-TIMP-3 with the truncated MMP cleavage site in between the sequences for LAP and TIMP-3 using primers 1 and 4. This LAP-TIMP-3 PCR products was then ligated into pcDNA6 restricted with *Eco*RI and *Xba*I restriction enzymes to generate LAP-TIMP-3 with the MMP clevage site PLGL. Human TIMP-3 without LAP was also cloned in pcDNA-6 downstream of the IL-2 signal sequence and with an additional N-terminal leucine residue using primers 5 (forward primer) and 4 (reverse primer, Table S1). The correct sequence of all constructs was verified by DNA sequencing (Eurofins Genomics, Ebersberg, Germany).

**Culture and transient transfection of HEK 293T cells**

293T cells were routinely cultured in DMEM (Sigma) supplemented with 10% FCS and penicillin and streptomycin. Transient transfections were performed using 25-kDa linear polyethylenimine (PEI) (Polysciences Inc. PA, USA). Stock solutions of PEI were prepared in water at a concentration of 1 mg/ml, and the pH adjusted to 7.0. HEK 293T cells were plated at a density of 2 x 10^6^ cells/plate in 10cm^3^ plates and transfected with 25 μg of DNA. The transfection complex was formed at a DNA:PEI ratio of 1:3 in serum-free OPTI-MEM, with a 30 minute incubation at room temperature prior to addition to the cells. After 24 h incubation at 37°C, 5% CO_2_, culture media were replaced with 5 mL of serum-free DMEM. NaClO_3_ was added to the serum-free media at a final concentration of 30 mM to help prevent endocytosis of the recombinant TIMP-3 after secretion. Cells were cultured for a further 48 h before collecting cell-conditioned media containing recombinant proteins.

**Purification of recombinant proteins from HEK293T conditioned media**

Cell supernatants from HEK 293T cells transiently transfected with pcDNA6-LAP-TIMP-3 were centrifuged to remove cell debris, dialysed exhaustively against PBS and applied to a cobalt-based chromatography resin (HiTrap Talon column; GE Healthcare, Buckinghamshire, UK). Columns were washed extensively with wash buffer (PBS pH 7.4, 300 mM NaCl, 0.01% Tween20, 50 mM imidazole) and eluted with elution buffer (PBS pH 7.4, 300 mM NaCl, 0.01% Tween20, 300 mM imidazole). Purification of L-TIMP-3 (i.e. TIMP-3 without LAP) was performed by applying HEK 293T cell conditioned media to anti-FLAG agarose (Sigma) without prior dialysis and eluting bound proteins using FLAG peptide at a final concentration of 200 µg/mL in FLAG buffer (50 mM Tris.HCl, pH 7.5, 150 mM NaCl, 10 mM CaCl_2_, 0.02% NaN_3_, 0.05% Brij 35). Eluted proteins were buffer exchanged into MMP cleavage buffer (50 mM Tris.HCl pH 7.4; 150 mM NaCl, 10 mM CaCl_2,_ 50 µM ZnCl_2,_ 0.05% Brij- 35) using a PD10 desalting column (GE Healthcare) and stored at -80°C until further use.

**Cleavage of LAP-TIMP-3 with MMP-1 or synovial fluid**

HEK 293T conditioned medium or purified proteins were treated with 0.2 µM of recombinant MMP-1 (Abcam, Cambridge, UK) and incubated for 18 hr at 37°C. In other experiments, purified LAP-TIMP-3 was incubated with synovial fluid from patients with osteoarthritis and was performed using 20% (vol/vol) of synovial fluid or patient sera with 80% supernatant (vol/vol) of LAP-TIMP-3 incubated at 37°C for 18 h. Cleavage of the LAP fusion protein by MMP was analysed by Western blotting by applying treated protein directly to acrylamide gels. Application of the samples containing synovial fluids or sera directly to acrylamide gels resulted in significant cross-reactivity of the samples with the antibodies used for the Western blot. Therefore, the TIMP-3 protein was immunoprecipitated from the protein/synovial fluid mixture using anti-FLAG magnetic beads prior to loading on acrylamide gels to allow analysis by Western blot. Proteins bound to the anti-FLAG magnetic beads could not be efficiently eluted using FLAG peptide up to 500 ug/mL, so for these immunoprecipitations of LAP-TIMP-3 from mixtures of protein and synovial fluid or sera, elution was performed using 0.1 M glycine pH 3.0 followed by immediate neutralisation with Tris.HCl, pH 9.

**Western Blotting**

HEK 293T cell culture supernatants or purified proteins were applied to 12% acrylamide SDS gels and transferred to nitrocellulose membranes (Millipore, Watford, UK) by electroblotting. After blocking in 5% dried skimmed milk in PBS containing 0.05% Tween 20, membranes were probed with monoclonal mouse anti-FLAG antibody, Clone M2 (Sigma) diluted 1 in 1000 or rabbit anti-TIMP-3 antibody (Abcam) diluted 1 in 1000 followed by detection with anti-rabbit-IgG-horseradish peroxidase conjugate (Sigma) or anti-mouse IgG horseradish peroxidase conjugate (Stratagene) both diluted 1 in 20,000. In some experiments, the fluorescently labelled secondary antibodies anti-mouse-IgG-BP-CFL 790 or anti-rabbit- IgG-CFL 790 (Santa Cruz Biotechnology, Inc) both diluted 1 in 20,000 were used to allow analysis by LiCOR imaging. Western blots were developed using advanced chemiluminescence (ECL) reagents (GE Healthcare) and exposed to autoradiography using Hyperfilm (GE Healthcare). Films were developed using an AGFA Curix 60 developer (Agfa Healthcare, Middlesex, UK). In some experiments, blots were analysed using a LiCOR Odyssey Fc imaging system (LiCOR Biosciences, Cambridge, UK).

**TIMP-3 ELISA**

The concentration of TIMP-3 expressed by HEK 293T cells was determined in HEK cell supernatants or lysed cells using a commercially available ELISA kit (R&D Systems, Minneapolis) according to the manufacturer’s instructions. All samples were measured in quadruplicate and optical density determined using a spectrophotometric ELISA plate reader (BioTek, Swindon, UK) and analyzed using Gen5 software V2.6.

**Activity assays for ADAMTS-4, MMP-1 and ADAM17**

The activity of ADAMTS-4 was monitored using the fluorescent peptide substrate carboxyfluorescein-Ala-Glu~Leu-Asn-Gly-Arg-Pro-Ile-Ser-Ile-Ala-Lys-N,N,N’,N’-tetramethyl-6-carboxyrhodamine (ADAMTS-4 and fluorescent peptide both generous gifts from Dr. L. Troeberg, University of Oxford, UK) at a final concentration of 0.5 μM with an excitation wavelength of 485 nm and an emission wavelength of 538 nm. Activity assays for MMP-1 and ADAM17 were purchased from Enzo Life Sciences (Exeter, UK) and used according to the manufacturer’s instructions. Both assays are also based on quenched fluorogenic peptides. Measurements were done in duplicate or triplicate.

**Release of glycosaminoglycans (GAG) from bovine cartilage explants**

Bovine articular cartilage from metacarpophalangeal joints was dissected into small shavings approximately 4-5 mm long and 2-3 mm wide. After dissection, the cartilage was allowed to rest for 24 h at 37°C, 5% CO_2_ in 1 mL of OPTI-MEM in a 48-well plate. The medium was then refreshed and the cartilage was rested for a further 48 h. Explants were then treated with 1 µM ADAMTS-4 that was pre-incubated for 1 hour at 37°C ± purified LAP-TIMP-3 ± 0.2 µM MMP-1 in 0.5 mL of fresh OPTI-MEM. After 3 days, the conditioned media were harvested and analysed for GAG release into the conditioned medium measured using a modification of the DMMB assay as described^[^[^3^](#_ENREF_3)^]^. Shark chondroitin sulphate was used as the standard on each plate. The percentage of total GAG released into the medium was calculated as follows: % of total GAG released = (total GAG in the medium)/(total GAG in the medium + total GAG remaining in the cartilage). Explants were digested with papain by incubation in 0.5 mL of digest buffer (50 mM sodium phosphate pH 6.5; 2 mM EDTA; 5 mM N-acetyl cysteine; 10 µg/mL papain) for 4 h at 65°C before assayed GAG as described above. All measurements were performed in triplicate.

| **Primer name** | **Primer sequence** |
| --- | --- |
| Primer 1 (F) | 5’ ctag *gaattc* c **CACCACCATCACCACCAT** TCC ACC AGC AAG ACC ATC GAC ATG 3’ |
| Primer 2 (R) | 5’ggg gtg gct ggg cga gca tgt gca AAG CCC GAG CGG GGA ACC GCC TCC 3’ |
| Primer 3 (F) | 5’GGA GGC GGT TCC CCG CTC GGG CTT tgc aca tgc tcg ccc agc cac ccc 3’ |
| Primer 4 (R) | 5’catg *tctaga* G **CTA CTT ATC GTC GTC ATC CTT GTA ATC** ggg gtc tgt ggc att gat gat gct 3’ |
| Primer 5 (F) | 5’ ctag *gaattc* c CTT tgc aca tgc tcg ccc agc cac ccc 3’ |

**Table S1.** Primer sequences used to clone LAP-TIMP-3 or L-TIMP-3. Bases in italics are restriction sites for *Eco*RI or *Xba*I. Sequences in upper case are sequences from mouse LAP and lower case from human TIMP-3. Sequences for tags for purification (His or FLAG) are in bold upper case. (F – forward primers; R – reverse primers).

**Supplementary Data References**

1. **Mullen L, Adams G, Foster J, Vessillier S, Koster M, Hauser H, Layward L, Gould D, Chernajovsky Y.** A comparative study of matrix metalloproteinase and aggrecanase mediated release of latent cytokines at arthritic joints. *Ann Rheum Dis*. 2014; 73: 1728-36.

2. **Troeberg L, Fushimi K, Scilabra SD, Nakamura H, Dive V, Thogersen IB, Enghild JJ, Nagase H.** The C-terminal domains of ADAMTS-4 and ADAMTS-5 promote association with N-TIMP-3. *Matrix Biol*. 2009; 28: 463-9.

3. **Gendron C, Kashiwagi M, Hughes C, Caterson B, Nagase H.** TIMP-3 inhibits aggrecanase-mediated glycosaminoglycan release from cartilage explants stimulated by catabolic factors. *FEBS Lett*. 2003; 555: 431-6.
