# Supplementary material for: Engineering of TIMP‐3 as a LAP‐fusion protein for targeting to sites of inflammation
Source: J Cell Mol Med. 2018 Nov 18;23(2):1617–21. doi: 10.1111/jcmm.14019 (PMC6349231; doi:10.1111/jcmm.14019)
Supplement: Supplementary file 4 [file JCMM-23-1617-s004.docx]

**Supplementary Material 2**

**Supplementary Figure Legends**

**Supplementary Figure 1**: LAP-TIMP-3 is cleaved by recombinant MMP-1 or synovial fluids (SF) from OA joints. LAP-TIMP-3 was immunoprecipitated from the protein/SF mixtures using anti-FLAG agarose prior to loading on gels to avoid cross-reactivity with the antibodies used for the Western blot. A. Western blot analysis of LAP-TIMP-3 incubated with MMP-1 or synovial fluids (SF) from 7 different patients with OA using anti-TIMP-3 antibody. B. Western blot analyses of a further 6 SFs from patients with OA using anti-FLAG antibody. The signal detected on Western blots was always greater for the LAP-TIMP-3 protein than for TIMP-3 without the LAP and this was the case for both anti-TIMP-3 and anti-FLAG antibodies.

**Supplementary Figure 2:** Antioxidant enzymes are not latent when expressed as LAP-fusion proteins. Measurement of reactive oxygen species (ROS) in NRK-52E (normal rat kidney) cells by ROS-Glo assay. Paraquat induces ROS in these cells and this is reduced in the presence of either LAP-ecSOD or LAP-catalase. Treatment of the LAP proteins with MMP did not affect activity of these antioxidant enzymes indicating lack of latency of these LAP-fusion proteins. This is likely due to the fact that the substrates for these enzymes are soluble molecules (H_2_O_2_ or O_2_^2-^) rather than cellular receptors.
